# Enhanced Imaging in Bladder Cancer: Fluorescence Cystoscopy and Molecular Diagnostics

**DOI:** 10.3390/life16050828

**Published:** 2026-05-16

**Authors:** Dominik Godlewski, David Aebisher, Dorota Bartusik-Aebisher, Klaudia Dynarowicz, Barbara Smolak, Magdalena Krupka-Olek, Aleksandra Kawczyk-Krupka

**Affiliations:** 1 Medical Centre in Łancut, 37-100 Łancut, Poland; remediumplus@o2.pl; 2 Department of Photomedicine and Physical Chemistry, Faculty of Medicine, Collegium Medicum, University of Rzeszów, 35-959 Rzeszów, Poland; 3 Department of Biochemistry and General Chemistry, Faculty of Medicine, Collegium Medicum, University of Rzeszów, 35-959 Rzeszów, Poland; dbartusikaebisher@ur.edu.pl (D.B.-A.); kdynarowicz@ur.edu.pl (K.D.); 4 Department of Diagnostic Imaging and Nuclear Medicine, Faculty of Medicine, Collegium Medicum, University of Rzeszów, 35-310 Rzeszów, Poland; bsmolak@ur.edu.pl; 5 Department of Internal Medicine, Angiology, and Physical Medicine, Center for Laser Diagnostics and Therapy, Medical University of Silesia in Katowice, Batorego Street 15, 41-902 Bytom, Poland; magda.krupka94@gmail.com

**Keywords:** bladder cancer, fluorescence cystoscopy, PDD, NBI, molecular diagnostics, biomarkers, TURBT

## Abstract

Background/Objectives: Bladder cancer remains one of the most frequently diagnosed malignancies worldwide and is characterized by high recurrence rates requiring long-term surveillance. Conventional white-light cystoscopy (WLC) remains the standard diagnostic method; however, it may fail to detect flat lesions such as carcinoma in situ or small papillary tumors. In recent years, enhanced imaging techniques, including fluorescence cystoscopy and autofluorescence-based systems, have been introduced to improve diagnostic accuracy. The aim of this study is to evaluate the usefulness of fluorescence-based diagnostic techniques and autofluorescence imaging supported by numerical color value (NCV) analysis in the detection and assessment of bladder lesions. Methods: The study was conducted at the Center of Photodynamic Diagnostics and Therapy, Department of Internal Medicine, Angiology and Physical Medicine, Medical University of Silesia in Bytom. Bladder mucosa was assessed using the Onco-LIFE optical imaging system, which enables visualization under both white-light and autofluorescence conditions. The study included 30 patients diagnosed with non-muscle-invasive bladder cancer or suspected bladder lesions, who underwent cystoscopic evaluation using white-light cystoscopy and autofluorescence imaging. From this cohort, three representative cases were selected for detailed qualitative presentation to illustrate different pathological conditions of the bladder mucosa. In selected cases, photodynamic diagnosis (PDD) using intravesical administration of 5-aminolevulinic acid (ALA) was performed prior to cystoscopic examination. Autofluorescence signals were analyzed using red and green fluorescence channels, and tissue characteristics were evaluated using the numerical color value parameter. Results: Representative cases of non-muscle-invasive bladder lesions were analyzed and compared using conventional white-light cystoscopy and autofluorescence imaging. The use of fluorescence-based imaging enabled improved visualization of suspicious mucosal changes compared with standard WLC. Differences in fluorescence patterns were observed between malignant lesions, inflammatory changes, and carcinoma in situ. NCV analysis allowed quantitative assessment of fluorescence signals and supported differentiation of pathological tissue from normal bladder mucosa. Conclusions: Fluorescence cystoscopy and autofluorescence-based imaging systems represent valuable tools for improving the detection of bladder lesions during endoscopic examination. The integration of enhanced optical imaging techniques with quantitative fluorescence analysis may increase diagnostic sensitivity and support targeted biopsy and tumor resection. Continued technological development and clinical experience may further expand the role of fluorescence diagnostics in the early detection and management of bladder cancer.

## 1. Introduction

Bladder cancer remains one of the most frequently diagnosed malignancies worldwide and is associated with substantial morbidity and mortality. Despite advances in oncological diagnostics and therapy, it continues to represent a significant clinical and public health challenge [1,2,3].

The burden of bladder cancer is currently highest in highly developed regions of the world [1,2,3,4,5]. Early detection remains crucial for improving patient survival and reducing recurrence rates. Bladder cancer is characterized by a high recurrence rate and the need for long-term surveillance, placing a considerable burden on healthcare systems and affecting patient quality of life. In particular, non-muscle-invasive bladder cancer (NMIBC) requires repeated cystoscopic examinations during follow-up, making accurate and sensitive diagnostic methods essential for effective disease management.

Modern diagnostic methods used to detect bladder cancer include imaging techniques such as ultrasound, intravenous urography (IVU), computed tomography (CT), and magnetic resonance imaging (MRI), as well as cystoscopy, biopsy, and urine cytology. In the initial evaluation of patients with suspected bladder cancer, ultrasound examination of the kidneys and urinary bladder is often used as a first-line imaging modality [6,7,8,9]. In addition, numerous urinary biomarkers have been developed to support the detection and monitoring of non-muscle-invasive bladder cancer. Many of these biomarkers demonstrate higher sensitivity than urinary cytology, although their specificity remains lower. While this has proven useful in certain clinical scenarios, most of these markers have not yet been incorporated into standard diagnostic guidelines [10]. Fluorescence-based urinary biomarkers represent a promising direction for non-invasive bladder cancer diagnostics [11,12].

Nevertheless, cystoscopy remains the gold standard for the diagnosis and management of bladder cancer. Conventional white-light cystoscopy allows direct visualization of the bladder mucosa and enables tissue sampling through biopsy. However, this technique has important limitations, particularly in the detection of flat lesions such as carcinoma in situ (CIS) and small papillary tumors. These diagnostic challenges have stimulated the development of enhanced imaging techniques designed to improve visualization of subtle mucosal abnormalities and increase the sensitivity of endoscopic examination.

One of the most widely used approaches is fluorescence cystoscopy, also referred to as photodynamic diagnosis [13]. Fluorescence-guided diagnostics is characterized by high sensitivity and specificity in detecting neoplastic lesions of the bladder mucosa [14,15]. Comparative studies have demonstrated that fluorescence cystoscopy increases the detection rate of carcinoma in situ by approximately 30% compared with conventional white-light cystoscopy [16]. Other optical imaging techniques have also been introduced to improve lesion visualization. Narrow Band Imaging (NBI) enhances vascular contrast and may improve the detection of suspicious lesions while reducing recurrence rates during follow-up. Similarly, blue-light cystoscopy (BLC) allows the detection of lesions that may be missed during standard cystoscopy, including up to 14% of Ta/T1 papillary tumors and approximately 40% of carcinoma in situ lesions [17]. Photodynamic diagnosis enables the visualization of neoplastic tissue at very early stages of development and facilitates more precise identification of malignant lesions during cystoscopic examination [18].

In recent years, growing interest has also focused on combining advanced imaging techniques with molecular diagnostics. The integration of optical imaging methods with targeted molecular probes and urinary biomarkers may further improve diagnostic accuracy and enable more individualized approaches to bladder cancer detection and surveillance.

The increasing use of fluorescence-based diagnostic techniques has led to the development of photosensitizing agents used in photodynamic diagnosis, most notably 5-aminolevulinic acid and hexyl aminolevulinate (HAL). 5-ALA acts as a precursor for the endogenous synthesis of protoporphyrin IX (PpIX), leading to selective accumulation of fluorescent porphyrins within malignant cells and enabling improved visualization of neoplastic tissue [19,20]. As a result, lesions that may be difficult to detect under white-light cystoscopy can be more easily identified under fluorescence conditions. Multiple studies have confirmed that 5-ALA fluorescence cystoscopy significantly improves the detection of malignant bladder lesions compared with standard cystoscopy [21,22,23,24,25,26].

A further development was the introduction of hexyl aminolevulinate (HAL) in 2003 [18]. HAL is a lipophilic derivative of 5-ALA characterized by improved cellular uptake and enhanced accumulation of photoactive porphyrins. Compared with 5-ALA, HAL allows the use of lower doses and shorter intravesical instillation times while maintaining high diagnostic performance [27,28]. Numerous randomized controlled trials have demonstrated that blue-light cystoscopy with HAL significantly improves the detection of bladder tumors. Data from a prospective multicenter registry conducted in the United States in 2018 demonstrated that BLC with HAL increased the detection rate of malignant lesions by 23%, papillary tumors by at least 12%, and carcinoma in situ by 43% compared with conventional white-light cystoscopy [29,30,31,32,33].

In addition to 5-ALA and HAL, other photosensitizing agents have been investigated for use in fluorescence-based diagnostics. One example is hypericin, a naturally occurring compound with strong photochemical activity [33,34,35]. Experimental studies suggest that hypericin demonstrates high sensitivity and specificity in the detection of bladder tumors [36,37,38]. However, its clinical applicability remains limited due to factors such as poor solubility.

Further research has explored the use of targeted molecular imaging agents, including carbonic anhydrase IX (CAIX), which plays an important role in tumor development and progression. One study demonstrated that CAIX-targeted imaging could distinguish malignant bladder tissue from benign tissue using blue-light cystoscopy, achieving sensitivity and specificity values of 88.0% and 93.75%, respectively. These findings suggest that CAIX-targeted molecular imaging may represent a promising approach for improving the accuracy of bladder cancer detection and guiding complete tumor resection [39,40,41].

Although numerous compounds have been investigated as potential photosensitizers, 5-aminolevulinic acid and hexyl aminolevulinate (HAL) remain the most widely used agents in clinical photodynamic diagnosis of bladder cancer today (Table 1).

To improve clarity and provide a structured synthesis of the available clinical evidence, the main findings of studies comparing white-light cystoscopy with fluorescence-based imaging techniques are summarized in Table 2.

Despite the growing availability of enhanced imaging techniques such as fluorescence cystoscopy and autofluorescence-based systems, several important limitations remain. In particular, the interpretation of fluorescence signals is often subjective and depends on operator experience, which may lead to variability in diagnostic accuracy and an increased rate of false-positive findings. Furthermore, although numerical color value analysis has been proposed as a quantitative tool to support autofluorescence imaging, its clinical applicability and ability to reliably differentiate between malignant, inflammatory, and normal bladder tissue remain insufficiently explored.

Therefore, there is a need for objective, reproducible methods that can support real-time decision-making during cystoscopic examination and improve the accuracy of lesion characterization. The present study aims to evaluate the usefulness of fluorescence-based diagnostic techniques combined with autofluorescence imaging and numerical color value analysis in the detection and assessment of bladder lesions, with particular emphasis on their potential to enhance diagnostic precision and support targeted biopsy.

## 2. Materials and Methods

### 2.1. Patients

This study was approved by the Bioethical Commission of the Silesian Medical University on the basis of Resolution No. KNW/022 and KB1/46/15 passed on 25 May 2015.

The study included 30 patients diagnosed with non-muscle-invasive bladder cancer or suspected bladder lesions, who underwent cystoscopic evaluation using white-light cystoscopy and autofluorescence imaging.

From this cohort, three representative cases were selected for detailed qualitative presentation to illustrate different pathological conditions of the bladder mucosa, including papillary tumor, carcinoma in situ, and inflammatory lesions. The analyzed cases were selected to represent different pathological conditions of the bladder mucosa, including papillary tumor, carcinoma in situ, and inflammatory lesions.

Inclusion criteria comprised patients undergoing diagnostic cystoscopy due to suspected bladder pathology. Patients with incomplete imaging data or inadequate visualization conditions were excluded from the analysis. All analyses were performed on the full cohort of 30 patients. Three representative cases were selected solely for illustrative purposes.

### 2.2. Apparatus/Equipment

Bladder imaging was performed using the Onco-LIFE optical imaging system (Xillix Technologies Corp., Richmond, BC, Canada), which enables endoscopic evaluation under both white-light and autofluorescence conditions.

The system utilizes blue-light excitation to induce autofluorescence of endogenous tissue fluorophores. Emitted fluorescence signals are captured and separated into two spectral channels: red (R; 650–700 nm) and green (G; 470–560 nm). These signals are subsequently processed to generate color-coded images that allow real-time visualization of tissue characteristics.

For quantitative analysis, fluorescence signals were digitally processed using dedicated Xillix software Design Suite 14.7. The red and green channels were isolated and converted into grayscale representations to enable objective assessment of fluorescence intensity. The numerical color value (NCV), defined as the ratio of red to green fluorescence signal, was used as a quantitative parameter to differentiate between normal and pathological bladder tissue.

This approach allowed both qualitative (visual) and quantitative (NCV-based) evaluation of bladder mucosa during cystoscopic examination.

### 2.3. ALA-PDD Procedure

Prior to the examination, all patients received local anesthesia. In the photodynamic diagnosis group, a buffered solution of 5-aminolevulinic acid at a concentration of 1.5 g/100 mL (Medac GmbH, Wedel, Germany) was instilled intravesically. The solution was retained in the bladder for approximately 2 h before the diagnostic procedure to allow sufficient accumulation of the photosensitizer in the urothelial tissue.

Following the incubation period, the bladder was rinsed with 0.9% NaCl solution, after which a fiberscope was introduced into the bladder to perform cystoscopic examination under the appropriate illumination conditions.

### 2.4. Statistical Analysis

Statistical analyses were performed on the full cohort of 30 patients. Statistical analyses were performed to evaluate relationships between the analyzed clinical variables. Fisher’s exact test was used to compare categorical variables. For numerical variables, nonparametric tests were applied, including the Mann–Whitney U test and the Kruskal–Wallis test, depending on the number of compared groups. The relatively small sample size (n = 30) limits statistical power.

A *p*-value of less than 0.05 was considered statistically significant. Linear regression analysis was performed to assess the relationship between numerical color value and selected clinical parameters, including tumor diameter, tumor type, and bladder capacity.

## 3. Results

### Patient Characteristics

A total of 30 patients were included in the analysis. Detailed qualitative presentation is provided for three representative cases. The observed lesions included different pathological conditions, allowing comparison of bladder mucosa under different imaging modalities. Autofluorescence imaging demonstrated improved visualization of suspicious lesions compared with white-light cystoscopy in the majority of cases. Differences in fluorescence patterns were observed between malignant lesions, inflammatory changes, and normal mucosa across the cohort. Quantitative analysis using numerical color value (NCV) showed higher values in malignant lesions compared with non-neoplastic tissue.

**Figure 1:**
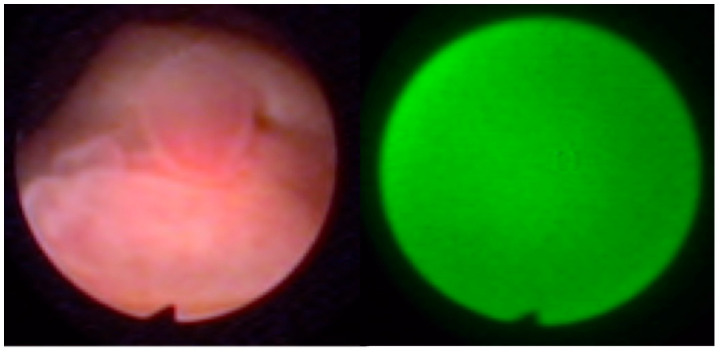
Bladder tumor visualized using white-light cystoscopy (**left**) and autofluorescence cystoscopy with numerical color value analysis (**right**). The lesion appears as a papillary structure under WLC, while increased red fluorescence intensity is observed in the corresponding area under autofluorescence imaging.

**Figure 2:**
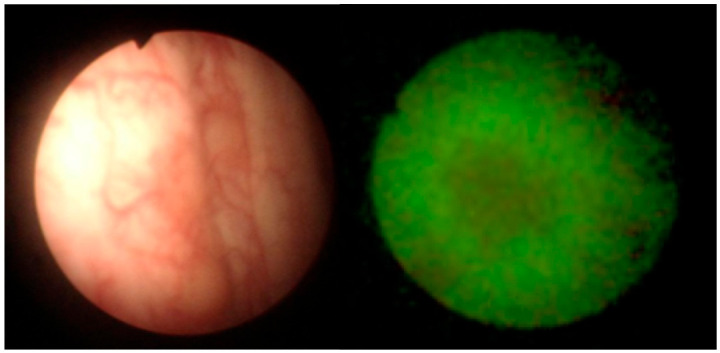
Recurrent transitional cell carcinoma visualized using white-light cystoscopy (**left**) and autofluorescence cystoscopy (**right**). Autofluorescence imaging enhances visualization of lesion boundaries compared with WLC.

**Figure 3:**
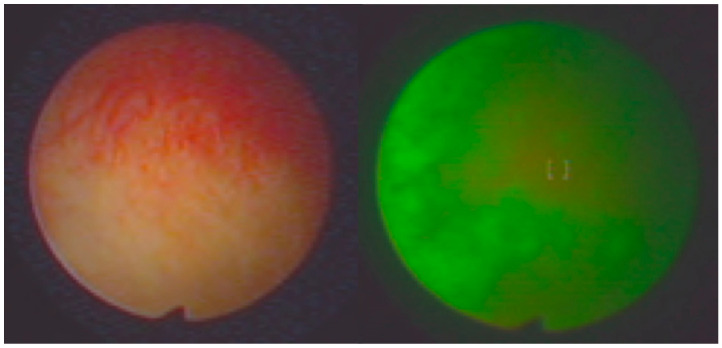
Chronic inflammatory lesion of the bladder mucosa visualized using white-light cystoscopy (**left**) and autofluorescence cystoscopy (**right**). Autofluorescence imaging shows heterogeneous fluorescence patterns in the affected area.

**Figure 4:**
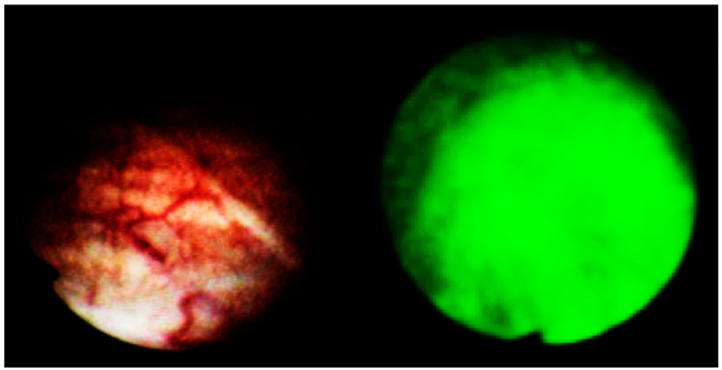
Carcinoma in situ visualized using white-light cystoscopy (**left**) and autofluorescence cystoscopy (**right**). The lesion is less distinct under WLC, while autofluorescence imaging highlights the affected mucosa with increased red fluorescence signal.

Representative endoscopic images obtained using both imaging modalities are shown in Figure 1, Figure 2, Figure 3 and Figure 4. The comparison illustrates differences in lesion visualization between standard white-light imaging and autofluorescence-based assessment.

In Figure 1, a bladder tumor is visualized under WLC and AFC/NCV conditions. Under white-light imaging, the lesion appears as a typical papillary structure, whereas autofluorescence imaging enhances lesion contrast, with the pathological area demonstrating increased red fluorescence intensity compared with surrounding tissue.

Figure 2 presents a case of recurrent transitional cell carcinoma. While the lesion is visible under WLC, autofluorescence imaging provides improved delineation of tumor margins, allowing more precise visualization of the extent of the pathological area.

Figure 3 shows chronic inflammatory changes in the bladder mucosa. Under autofluorescence conditions, these lesions demonstrate intermediate fluorescence patterns, which may partially overlap with neoplastic features. This finding highlights the potential limitation of autofluorescence imaging related to false-positive results in inflammatory conditions.

Figure 4 illustrates carcinoma in situ, which is typically difficult to detect using conventional white-light cystoscopy. In this case, autofluorescence imaging clearly enhances lesion visibility, with a marked increase in red fluorescence signal, facilitating identification of otherwise subtle mucosal abnormalities.

Across all analyzed cases, autofluorescence imaging enabled improved visualization of suspicious areas compared with WLC. Differences in fluorescence patterns were observed between malignant lesions, inflammatory changes, and normal mucosa. These variations were reflected in differences in numerical color value, supporting the potential role of NCV as a quantitative parameter in distinguishing pathological tissue from normal bladder mucosa.

## 4. Discussion

Cystoscopy remains the gold standard for the diagnosis of bladder cancer; however, its diagnostic accuracy under white-light conditions is limited, particularly in the detection of flat lesions such as carcinoma in situ and small papillary tumors. Fluorescence cystoscopy, also referred to as photodynamic diagnosis, has been developed to improve visualization of such lesions by exploiting differences in fluorescence properties between normal and pathological tissue [42,43,44].

**Figure 5:**
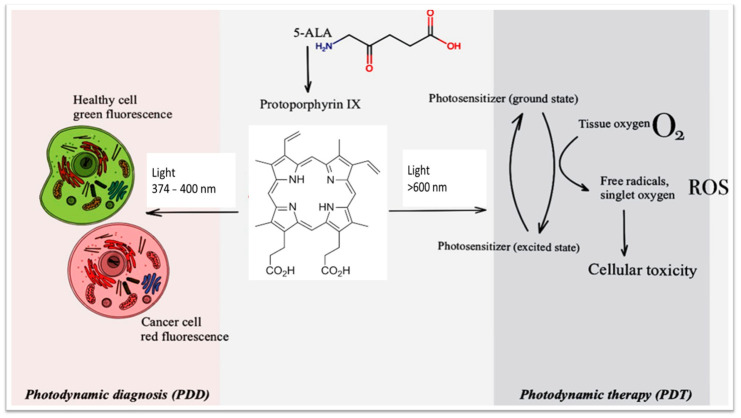
Scheme of therapy and photodynamic diagnostics of neoplasms.

In photodynamic diagnostics, a fluorophore is administered intravesically prior to the examination. The compound is absorbed by the urothelial cells and is preferentially metabolized by dysplastic or neoplastic cells. When exposed to light of an appropriate wavelength, these cells emit a characteristic red fluorescence that allows improved visualization of suspicious lesions. The most commonly used photosensitizers for PDD are 5-aminolevulinic acid and hexyl aminolevulinate, both of which demonstrate favorable safety profiles and clinical applicability [45,46,47]. The diagnostic principle of fluorescence cystoscopy is based on the intracellular accumulation of protoporphyrin IX, which emits red fluorescence under blue-light excitation, whereas normal urothelial tissue demonstrates weaker fluorescence signals, enabling visual differentiation between normal and pathological areas [48,49,50]. A schematic overview of photodynamic diagnostics and therapeutic workflow is presented in Figure 5.

In the present study, autofluorescence imaging supported by numerical color value analysis enabled improved visualization of bladder lesions compared with conventional white-light cystoscopy. Malignant lesions, including carcinoma in situ and recurrent transitional cell carcinoma, demonstrated increased red fluorescence intensity relative to normal bladder mucosa, which appeared predominantly green. These findings are consistent with previously described optical characteristics of neoplastic tissue and support the diagnostic value of fluorescence-based imaging [51,52]. Importantly, NCV analysis provided an additional quantitative parameter reflecting the ratio of red to green fluorescence signals, with higher NCVs observed in malignant lesions. This suggests that quantitative fluorescence assessment may complement visual interpretation and improve diagnostic confidence during cystoscopic examination [53]. Importantly, while three representative cases are presented in detail, the conclusions are based on analysis of the entire cohort.

In contrast, inflammatory lesions showed intermediate fluorescence characteristics, which may overlap with neoplastic patterns and contribute to potential false-positive findings. This observation is consistent with previous reports indicating that inflammation, previous intravesical therapy, or recent surgical intervention may influence autofluorescence signals and reduce specificity [54,55,56]. The variability of fluorescence patterns observed during autofluorescence cystoscopy may be attributed to several biological and technical factors, including inflammatory changes, increased vascularization, edema, and immune cell infiltration, all of which may alter tissue optical properties and lead to heterogeneous fluorescence patterns. Similarly, the presence of blood or microhemorrhages may absorb excitation light and reduce fluorescence intensity, leading to potential underestimation of pathological changes [57,58].

Another important factor is photobleaching, which refers to the gradual loss of fluorescence signal intensity during prolonged exposure to excitation light. This phenomenon may reduce lesion visibility and affect real-time interpretation during cystoscopic examination. Additionally, technical factors such as uneven bladder distension, angle of illumination, and distance between the endoscope and mucosa may further contribute to variability in fluorescence appearance [59]. From a clinical perspective, interpretation of fluorescence findings requires careful correlation with cystoscopic appearance and patient history. In cases where false-positive results are suspected, particularly in the presence of inflammation or recent surgical intervention, targeted biopsy remains essential for definitive diagnosis, and repeat cystoscopy or follow-up evaluation may be necessary to confirm the nature of suspicious lesions [60,61].

In clinical practice, several technical considerations must be taken into account during fluorescence cystoscopy. Proper bladder distension, adequate illumination, and maintenance of a clear visual field are essential to ensure optimal visualization and reduce false-positive findings. Bleeding should be minimized, as it may significantly affect fluorescence signal intensity. Suspicious areas should be biopsied promptly, as photobleaching may reduce fluorescence visibility over time [62,63]. An additional advantage of fluorescence cystoscopy is its ability to detect flat lesions such as carcinoma in situ, which are frequently missed during conventional white-light examination, with important clinical implications, particularly in patients with recurrent non-muscle-invasive bladder cancer [64].

The concept of optical biopsy is particularly relevant to autofluorescence-based systems such as Onco-LIFE, which enable both qualitative and quantitative assessment of bladder mucosa. These systems allow real-time analysis of fluorescence signals and may support differentiation between neoplastic, inflammatory, and normal tissue [65,66,67]. Bochynek et al. demonstrated that autofluorescence cystoscopy combined with NCV analysis may serve as a valuable tool for early detection of bladder lesions and for guiding targeted biopsies, with a reported correlation between NCVs and histopathological findings supporting the clinical utility of this approach [68].

Multiparametric cystoscopy represents an advanced diagnostic concept aimed at improving the detection and characterization of bladder lesions through the simultaneous use of multiple imaging modalities. By combining techniques such as white-light cystoscopy, blue-light cystoscopy, and autofluorescence imaging, clinicians may obtain more comprehensive information about bladder mucosa during endoscopic evaluation. Previous studies have demonstrated the feasibility of real-time multispectral imaging, with certain lesions detectable only when multiple imaging techniques are combined, highlighting the added diagnostic value of such approaches [69]. Similarly, combining white-light and blue-light cystoscopy has been shown to significantly improve diagnostic sensitivity, reaching approximately 98.5% in detecting malignant lesions [70].

Although multiparametric cystoscopy was not directly evaluated in the present study, the combination of autofluorescence imaging and NCV analysis used in our work may be considered a step toward such integrated diagnostic strategies. Future developments in bladder cancer diagnostics are likely to focus on the integration of molecular diagnostics with advanced imaging techniques. The use of molecularly targeted fluorescent probes and urinary biomarkers may enable more precise visualization of malignant tissue and support patient selection for enhanced diagnostic procedures such as photodynamic diagnosis or blue-light cystoscopy [71,72]. The combination of optical imaging with molecular diagnostics may therefore facilitate a more personalized approach to bladder cancer detection and surveillance, improving both diagnostic accuracy and clinical decision-making, with ongoing research aiming to integrate multiple diagnostic modalities within a single cystoscopic procedure [73,74].

## 5. Conclusions

Fluorescence-based cystoscopy, including autofluorescence imaging, improves the detection of bladder lesions compared with conventional white-light cystoscopy, particularly in cases of subtle or flat lesions such as carcinoma in situ.

In this study, autofluorescence imaging combined with numerical color value (NCV) analysis enabled both qualitative and quantitative assessment of bladder mucosa, supporting differentiation between malignant and non-malignant tissue. Increased red fluorescence intensity and higher NCVs were consistently observed in neoplastic lesions.

These findings suggest that NCV may serve as a useful adjunctive parameter to reduce subjectivity in fluorescence image interpretation and support real-time decision-making during cystoscopy.

However, overlap in fluorescence patterns between inflammatory and neoplastic lesions remains a limitation and may contribute to false-positive findings.

Overall, the integration of autofluorescence imaging with quantitative analysis represents a promising step toward more objective and precise optical diagnostics in bladder cancer, although validation in larger, prospective studies is required.

## Figures and Tables

**Table 1:** Selected photosensitizing agents used in photodynamic diagnosis of bladder cancer [20,27,28,36,37,38].

Photosensitizer	Mechanism	Clinical Status
**5-ALA**	Precursor of protoporphyrin IX accumulating in tumor cells	Used in fluorescence cystoscopy
**HAL**	Lipophilic derivative of 5-ALA with improved cellular uptake	Approved for blue-light cystoscopy
**Hypericin**	Natural photoactive compound with strong fluorescence properties	Experimental/investigational

**Table 2:** Summary of clinical outcomes of fluorescence-based imaging techniques compared with white-light cystoscopy.

Clinical Outcome	Technique	Key Findings	Limitations
**Detection of carcinoma in situ (CIS)**	5-ALA PDD/HAL-BLC	Significantly improved detection of flat lesions compared with WLC	Increased false-positive rate, especially in inflammatory lesions
**Detection of papillary tumors (Ta/T1)**	HAL-BLC	Improved detection of small papillary tumors	Reduced specificity in some cases
**Recurrence rate after TURBT**	PDD (5-ALA/HAL)	Reduced recurrence rates due to more complete tumor resection	Requires additional procedure (intravesical instillation)
**Overall tumor detection**	BLC/PDD	Higher sensitivity compared with WLC	Higher rate of false-positive findings
**Tissue characterization**	Autofluorescence (AFC)	Enables real-time differentiation using fluorescence patterns and NCV	Overlap between inflammatory and neoplastic lesions

## Data Availability

The data presented in this study are available on request from the corresponding author. The data are not publicly available due to ethical issues.
